# The inverse relationship between bladder and liver in 4-aminobiphenyl-induced DNA damage

**DOI:** 10.18632/oncotarget.2722

**Published:** 2014-12-17

**Authors:** Arup Bhattacharya, Joshua J. Klaene, Yun Li, Joseph D. Paonessa, Aimee B. Stablewski, Paul Vouros, Yuesheng Zhang

**Affiliations:** ^1^ Department of Chemoprevention, Roswell Park Cancer Institute, Buffalo, NY, USA; ^2^ Barnett Institute and Department of Chemistry and Chemical Biology, Northeastern University, Boston, MA, USA; ^3^ Department of Urology, Roswell Park Cancer Institute, Buffalo, NY, USA; ^4^ Department of Molecular & Cellular Biology, Roswell Park Cancer Institute, Buffalo, NY, USA

**Keywords:** Bladder cancer, 4-aminobiphenyl, DNA damage, UDP-glucuronosyltransferase

## Abstract

Bladder cancer risk is significantly higher in men than in women. 4-Aminobiphenyl (ABP) is a major human bladder carcinogen from tobacco smoke and other sources. In mice, male bladder is more susceptible to ABP-induced carcinogenesis than female bladder, but ABP is more carcinogenic in the livers of female mice than of male mice. Here, we show that castration causes male mice to acquire female phenotype regarding susceptibility of bladder and liver to ABP. However, spaying has little impact on organ susceptibility to ABP. Liver UDP-glucuronosyltransferases (UGTs) are believed to protect liver against but sensitize bladder to ABP, as glucuronidation of ABP and its metabolites generally reduces their toxicity and promotes their elimination via urine, but the metabolites are labile in urine, delivering carcinogenic species to the bladder. Indeed, liver expression of ABP-metabolizing human UGT1A3 transgene in mice increases bladder susceptibility to ABP. However, ABP-specific liver UGT activity is significantly higher in wild-type female mice than in their male counterparts, and castration also significantly increases ABP-specific UGT activity in the liver. Taken together, our data suggest that androgen increases bladder susceptibility to ABP via liver, likely by modulating an ABP-metabolizing liver enzyme, but exclude UGT as an important mediator.

## INTRODUCTION

Cancer incidence of the urinary bladder is nearly four times higher in men than in women in the United States [1–3]. A similar trend exists in many other countries as well [4]. In line with the gender disparity in bladder cancer incidence, the mortality rate of this disease is also more than three-fold higher in men than in women [5]. The molecular basis of the high male susceptibility to bladder cancer remains poorly understood, but many lines of evidence indicate that this is not due to exposure to bladder carcinogens. Aromatic amines from tobacco smoke and occupational exposure, 4-aminobiphenyl (ABP) in particular, are widely recognized as the main cause of bladder cancer. Levels of ABP-DNA adducts were found to be up to 8-fold higher in bladder specimens of smokers than in that of nonsmokers [6–8]. However, male and female mice exposed to aromatic amines under the same experimental condition recapitulate the human phenomenon. For example, when exposed to ABP at the same dose for the same time, bladder tumor developed in 20% of the male mice but in none of the female mice [9]. Moreover, similar gender disparity in bladder cancer risk was also observed in humans in the absence of tobacco smoking, exposure to occupational hazards and other risk factors [2, 3].

Several animal studies have shown that androgen and androgen receptor (AR) signaling promotes whereas estrogen inhibits bladder cancer development induced by *N*-butyl-*N*-(4-hydroxybutyl)nitrosamine (BBN) [10–12]. However, BBN is not a human bladder carcinogen. Lin et al. recently reported that constitutive β-catenin activation in mouse bladder epithelium resulted in male specific bladder cancer development and that AR was a critical mediator [13]. However, abnormal β-catenin activation appears to be rare in human bladder cancer [14]. Interestingly, in animals treated with aromatic amines, there is an inverse association of carcinogenesis between the bladder and liver. For example, in mice, while ABP was significantly more tumorigenic in the male bladder than in the female bladder as mentioned above, the gender disparity in tumor development in the liver in the same mice was reversed (33% in the female and none in the male) [9]. Similarly, in mice treated with 2-aminodiphenylene oxide, liver tumor incidence was significantly higher in the females, whereas bladder tumor incidence was significantly higher in the males [15]. Moreover, the ABP-DNA adduct levels formed in the bladders and livers of ABP-treated mice followed the same pattern of tumor development in these organs [16–19]. These results suggest that the liver may play an important part in the gender disparity of bladder cancer risk. Given that aromatic amines are mainly metabolized in the liver and the carcinogenic metabolites are delivered to the bladder via urinary excretion [20], it is conceivable that certain metabolic enzymes in the liver may mediate the dichotomy of carcinogenicity of aromatic amines in the bladder and liver. Liver cancer incidence in humans, however, is significantly higher in men than in women [1, 4, 21], but the main risk factors of human liver cancer, including chronic infection with hepatitis viruses, cirrhosis, and exposure to aflatoxins, may mask the potential adverse effects of aromatic amines in this organ.

Among the enzymes that are involved in the biotransformation of aromatic amines in the liver are UDP-glucuronosyltransferases (UGTs), a family of enzymes responsible for glucuronidation of a variety of substrates, including aromatic amines and their metabolites [20], such as UGT1A1, 1A3, 1A4, 1A9 and 2B7 [22–24]. While the glucuronides of aromatic amines and their metabolites that are formed in the liver may be relatively nonreactive and are rapidly excreted in urine, they are thought to pose a problem for the bladder, as the conjugates are labile, especially in acidic urine, thereby delivering carcinogenic species to the bladder [20]. Conceivably, higher liver UGT activity may provide more protection for the liver against aromatic amines but may render the bladder more susceptible to these compounds. This raises the question as to whether certain UGTs in the liver may be responsible at least in part for the gender disparity in bladder cancer risk. Here, we describe a study of potential involvement of androgen, estrogen and liver UGT in bladder and liver susceptibility to ABP-induced DNA damage in mice.

## RESULTS

### The impact of castration and spaying on the inverse association of ABP-induced DNA damage between bladder and liver

ABP is a major human bladder carcinogen from tobacco smoke and other sources [25, 26]. Approximately 80% of ABP-DNA adducts formed in human bladder cells and tissues are *N*-(deoxyguanosin-8-yl)-4-aminobiphenyl (dG-C8-ABP) [7, 27]. The dG-C8-ABP levels formed in the bladders and livers of mice exposed to ABP followed the same patterns of tumor development in these organs [28]. Higher levels of ABP-DNA adducts in human bladder tumors were also associated with more aggressive behavior of the tumors [29]. In the present study, dG-C8-ABP was used as a surrogate biomarker of bladder susceptibility to ABP carcinogenicity.

**Figure 1:**
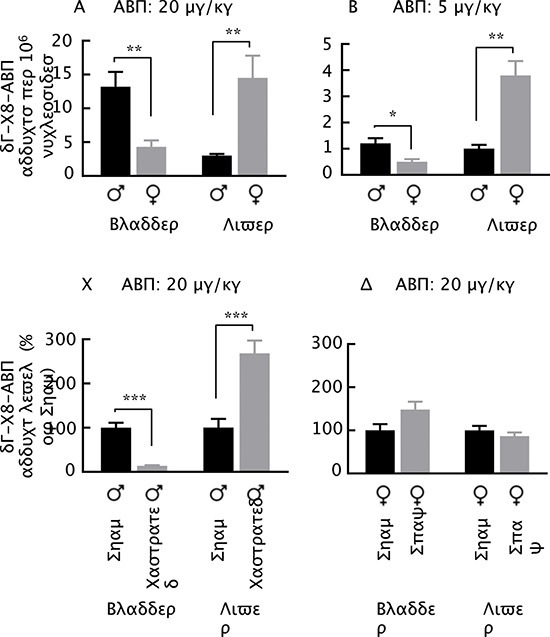
dG-C8-ABP levels in ABP-treated mice **(A, B)** Male and female mice at 7–8 weeks of age were given a single intraperitoneal dose of ABP. **(C)** Male mice were castrated or sham operated at 7–8 weeks of age; 3 weeks later, the mice were treated with a single intraperitoneal dose of ABP. **(D)** Female mice were spayed or sham operated at 4 weeks of age; 3–4 weeks later, the mice were treated with a single intraperitoneal dose of ABP. All mice were killed 24 h after ABP treatment for measurement of dG-C8-ABP in their bladders and livers. Each value is mean + SE (*n* = 4–8). *P < 0.05, **P ≤ 0.01, ***P < 0.001.

Background levels of dG-C8-ABP in the bladder and liver tissues of mice were below the detection limit of five adducts per 10^9^ nucleosides. However, there were about 13.2 dG-C8-ABP adducts per 10^6^ nucleosides in the bladders of male mice at 24 h after a single intraperitoneal dose of ABP at 20 mg/kg, while the adduct level in the female bladders was 3.1-fold lower (Fig. 1A). There were about 3.0 dG-C8-ABP adducts per 10^6^ nucleosides in the livers of ABP-treated male mice, whereas the adduct level was 4.8-fold higher in the female livers (Fig. 1A). A similar trend of gender disparity in susceptibility to ABP in the bladder and liver and of the inverse association between the bladder and liver was detected in mice treated with ABP at 5 mg/kg (Fig. 1B), although the absolute dG-C8-ABP levels in these tissues were markedly lower than that induced by ABP at 20 mg/kg. These results suggest that the liver may be involved in the gender-related bladder susceptibility to ABP, perhaps by modulating carcinogen delivery to the bladder via metabolic disposition in urine.

Next, male mice were castrated or sham operated, and 3–4 weeks later the mice were treated with ABP at 20 mg/kg for 24 h, followed by measurement of dG-C8-ABP in the bladders and livers. Castration reduced the level of bladder dG-C8-ABP by 7.4-fold but increased the level of liver dG-C8-ABP by 2.7 fold. Thus, castration apparently causes the male mice to adopt the phenotype of female mice in response to ABP. Given that castration reduces bladder susceptibility to ABP at the cost of more liver DNA damage and that liver is the main organ of ABP bio-activation, it seems likely that androgen may sensitize bladder to ABP at least partly by modulating liver metabolism of ABP and delivery of its carcinogenic metabolites to bladder. We also examined the potential effect of estrogen on the susceptibility of bladder and liver to ABP. Female mice were spayed or sham operated, and 3–4 weeks later the mice were treated with ABP at 20 mg/kg for 24 h. As shown in Fig. 1D, the impact of spaying on the susceptibility of bladder and liver to ABP is statistically insignificant, although it appears to be opposite to that of castration. Thus, estrogen does not appear to be significantly involved in the gender-related inverse association of organ susceptibility to ABP between bladder and liver.

### The effects of castration and spaying on liver UGT activity

**Figure 2:**
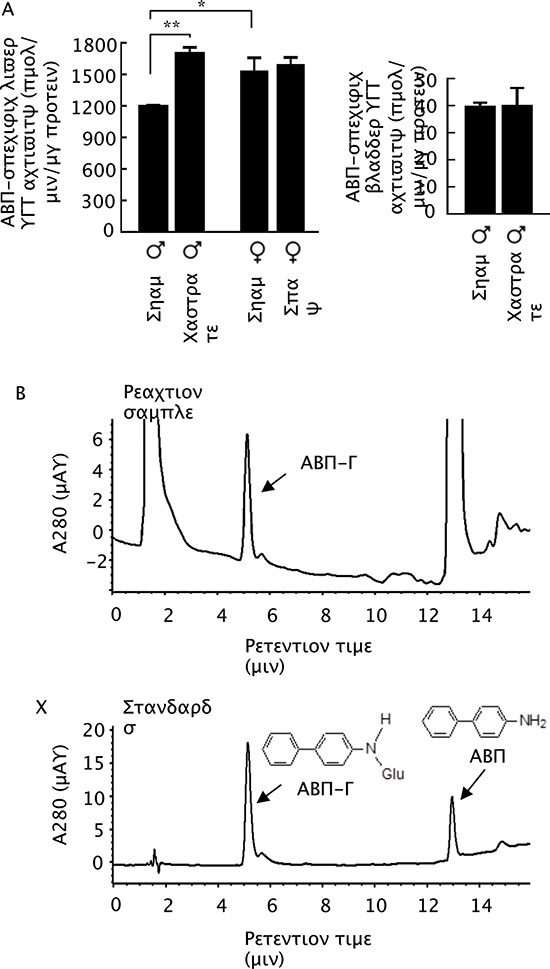
ABP-specific UGT activity in bladder and liver **(A)** Castration and sham operation were performed when mice were 7–8 weeks of age, and bladders and livers were collected from the mice at 11–12 weeks of age. Spaying and sham operation were performed when mice were 4 weeks of age, and bladders and livers were collected from the mice at 7–8 weeks of age. Each value is mean + SE (*n* = 3). *P < 0.05, **P < 0.01. **(B)** An example of HPLC measurement of ABP-G formed in reaction catalyzed by UGT in bladder and liver samples. **(C)** HPLC profiles of pure ABP-G and ABP.

The inverse association of ABP-induced DNA damage between bladder and liver and the reversing impact of castration on such association, already described above, seems to fit with a mechanism mediated by liver UGT. As mentioned before, liver UGT is believed to protect liver against the toxicity of aromatic amines by converting these compounds and their metabolites to glucuronides but to promote bladder exposure to the carcinogens, because the glucuronides are disposed in the urine but are unstable and dissociate to carcinogenic species. ABP glucuronidation activity in the liver tissues of male and female mice could be readily measured, but unexpectedly, the activity was 1.3-fold higher in the female liver than in the male liver in the sham mice (Fig. 2A, same finding in mice with no surgery). The metabolite formed in the enzymatic reaction by liver UGT was the glucuronide in which the glucuronic acid is attached to the nitrogen atom of ABP (APB-G) (Fig. 2B), which also co-eluted in HPLC with the synthetic standard (Fig. 2C). This is a well-known ABP metabolite generated by UGT [20]. Moreover, in castrated mice, liver ABP-specific UGT activity increased 1.4-fold, compared to that in sham-operated male mice (Fig. 2A). Thus, androgen may significantly suppress ABP glucuronidation in the liver. In contrast, estrogen does not appear to significantly modulate liver ABP-specific UGT activity, as there was little difference between spayed and sham operated female mice (Fig. 2A). Notably, our attempt to measure levels of the glucuronic acid conjugate of ABP in urine of ABP-dosed mice was unsuccessful, as the conjugate dissociated to ABP during urine collection. We also measured ABP-specific UGT activity in the bladder, but castration did not significantly impact the activity in this organ (Fig. 2A).

### UGT1A3 expression in transgenic mice and tissue ABP-specific glucuronidation activity

**Figure 3:**
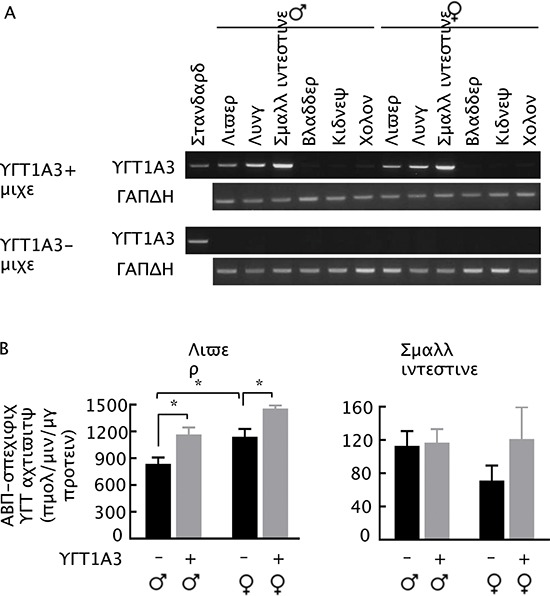
UGT1A3 expression in homozygous UGT1A3 transgenic mice (UGT1A3+) **(A)** RT-PCR analysis of UGT1A3 in various organs of UGT1A3+ mice at 7–8 weeks of age, using age-matched non-transgenic mice (UGT1A3-) as control. **(B)** ABP-specific UGT activity in organs of UGT1A3+ mice and UGT1A3- mice at 7–8 weeks of age. Each value is mean + SE (*n* = 3) *P < 0.05.

Our surprising finding that higher liver UGT activity towards ABP is associated with a higher level of dG-C8-ABP in the liver but a lower level of dG-C8-ABP in the bladder in mice, as described above, raises the unsettling question of whether liver UGT sensitizes liver to but protects bladder against ABP. To better understand the role of liver UGT, we generated transgenic mice expressing human UGT1A3. UGT1A3 was chosen for the study, because it is known to catalyze the glucuronidation of ABP and other aromatic amines [24], but is a pseudogene in mice [30]. The transgene was driven by a chimeric promoter composed of minimal mouse albumin promoter and the mouse alpha fetoprotein enhancer II, which was intended for liver-specific expression of the transgene. UGT1A3 was undetectable in the tissues of non-transgenic littermates, as measured by RT-PCR, but unexpectedly, UGT1A3 appeared to be expressed higher in the small intestine and lung than in the liver in both male and female transgenic mice, although it was not detected in the bladder and other tissues (Fig. 3A). Clearly, transgene expression was not as specific as was initially thought. However, while ABP-specific UGT activity increased significantly in the livers of both male and female transgenic mice (Fig. 3B), consistent with UGT1A3 mRNA expression, ABP-specific UGT activity in the small intestines of the transgenic mice, regardless of gender, was not significantly different from that in the non-transgenic littermates (Fig. 3B), suggesting a lack of significant UGT1A3 protein expression in this organ. The ABP-specific UGT activity in the small intestine was also approximately 10-fold lower than in the liver. ABP-specific UGT activity was below the detection limit of 10 pmol/min/mg microsomal protein in the lungs of both transgenic mice and non-transgenic littermates. These results are also consistent with the understanding that liver is the primary metabolic organ for ABP and other aromatic amines.

### Bladder and liver susceptibility to ABP in UGT1A3 transgenic mice

**Figure 4:**
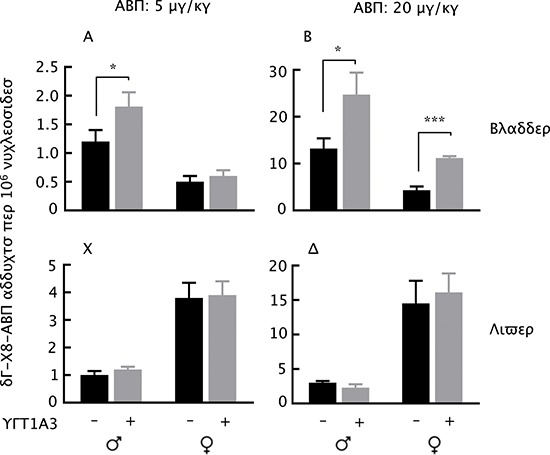
dG-C8-ABP levels in ABP-treated homozygous UGT1A3 transgenic mice (UGT1A3+) and age-matched non-transgenic littermates (UGT1A3–) Mice at 7–8 weeks of age were treated with a single intraperitoneal dose of ABP at 5 or 20 mg/kg; 24 h later, the mice were killed and levels of dG-C8-ABP were measured in the bladders and livers. Each value is mean + SE (*n* = 3–4). *P < 0.05, ***P < 0.001.

The UGT1A3 transgenic mice and their littermates were given a single intraperitoneal dose of ABP at 5 or 20 mg/kg; 24 h later the mice were killed, and dG-C8-ABP levels in the bladder and liver were measured. As expected, ABP caused a dose-dependent increase in the levels of bladder dG-C8-ABP, and the adduct levels were uniformly higher in the male mice than in the female mice, but were also significantly higher in the transgenic mice than in their non-transgenic littermates, regardless of gender, except in the bladders of female mice treated with ABP at 5 mg/kg, where the difference was not statistically significant (Fig. 4A & 4B). Specifically, in mice treated with ABP at 5 mg/kg, the level of bladder dG-C8-ABP in male homozygous UGT1A3 transgenic mice was 1.5-fold higher than in the male non-transgenic littermates, while the difference in the female mice was not statistically significant between the two genotypes (Fig. 4A). In mice treated with ABP at 20 mg/kg, the level of bladder dG-C8-ABP in the homozygous UGT1A3 transgenic mice was 1.9-fold higher in the male and 2.6-fold higher in the female than in the non-transgenic littermates (Fig. 4B).

Although the level of dG-C8-ABP in the female livers were uniformly higher than in the male livers, regardless of UGT1A3 genotype, as expected, we did not detect a significant impact of UGT1A3 genotype on dG-C8-ABP formation in the liver (Fig. 4C & 4D).

## DISCUSSION

While androgen and AR have been previously shown to promote bladder tumorigenesis induced by BBN, as mentioned before, to the best of our knowledge, the present study is the first to show the potential role of androgen in sensitizing male bladder to a human bladder carcinogen, i.e., ABP. Moreover, our results suggest that androgen functions via the liver to exert its impact on bladder susceptibility to ABP. We show that the potential promoting effect of androgen on ABP-induced DNA damage in the bladder is accompanied by reduced DNA damage by ABP in the liver, whereas the marked decrease in bladder DNA damage caused by ABP in castrated mice is associated with a marked increase in liver DNA damage in the same mice. The effect of androgen on bladder and liver susceptibility to ABP is likely mediated by one or more ABP-metabolizing enzymes in the liver, as ABP and other aromatic amines are not carcinogenic themselves but undergo metabolic transformation in the liver. In contrast, our results show that estrogen does not significantly modulate bladder and liver susceptibility to ABP.

Biotransformation of aromatic amines in liver is complex and includes hydroxylation by cytochrome P450 enzymes, acetylation by arylamine acetyltransferases, glucuronidation by UGT, and sulfation by sulfotransferases [20]. UGT seemed to be a likely mediator for the androgen-driven dichotomy of ABP-induced DNA damage in bladder and liver. As mentioned before, liver UGT is believed to protect the liver by catalyzing glucuronidation of ABP and its metabolites, but the metabolites, once excreted in the urine, are readily dissociated, posing a risk for the bladder. However, our experiment shows that liver UGT activity towards ABP is significantly lower in male mice than in female mice and that castration in male mice significantly increases liver UGT activity towards ABP. These results show that androgen potentially suppresses the expression of ABP-metabolizing UGT in the liver and rules out liver UGT as a sensitizer of bladder damage by ABP. Notably, our result differs from a previous finding that overall ABP *N*-glucuronidation activity in male mouse liver samples is significantly higher than in their female counterparts [17], even though the same strain of mice were used in both studies. However, the glucuronidation product is somewhat unstable and precautions need to be taken for accurate measurement. Our result is consistent with a recent study showing that many UGT isoforms in cultured human bladder epithelial cells and mouse bladder tissues are down regulated by androgen-mediated AR signaling [31]. Moreover, several Ugt isoforms, including Ugt1a1, Ugt1a5, Ugt1a9 and Ugt1a10, are expressed at higher levels in the livers of female mice than in their male counterparts [32], and at least Ugt1a1 and 1a9 are believed to be active towards ABP and other aromatic amines [22, 23]. However, while liver UGT does not account for the gender-related inverse association of ABP-induced DNA damage in the bladder and liver, our experiment with UGT1A3 transgenic mice indicates that liver UGT does enhance bladder exposure to ABP, likely by promoting delivery of carcinogenic metabolites of ABP to bladder.

The androgen-regulated liver factor(s) that is responsible for the gender disparity of bladder susceptibility to ABP remains to be identified, but is likely to be an ABP-metabolizing enzyme. However, knockout of cytochrome P450 Cyp1a2, a major metabolic activator of ABP, had little effect on ABP-induced DNA damage in the bladders and livers in mice [18]. Our finding that this liver factor is potentially suppressed by androgen but is not affected by estrogen may facilitate the search for it. We have previously shown that knockout of Nrf2, a major regulator of cytoprotective and drug-metabolizing genes, reduces the level of dG-C8-ABP in the bladder but increases its level in the liver in mice treated with ABP [33]. Thus, androgen and Nrf2 seem to behave similarly with regard to modulating bladder and liver susceptibility to ABP. Could androgen and Nrf2 target the same ABP-metabolizing enzyme in the liver? Also, in the present study, we focused on ABP and used dG-C8-ABP as a surrogate of ABP carcinogenicity. Further investigation of gender disparity in bladder cancer risk with other aromatic amines or using tumor development as an end point is also important.

## MATERIALS AND METHODS

### Reagents

ABP and ABP-*N*-glucuronide (ABP-G) standard were purchased from Sigma (St Louis, MO) and Toronto Research Chemicals (Toronto, Ontario), respectively.

### Castration and spaying of mice

For castration, both testes were removed from 7–8 week old male C57BL/6N mice (Taconic, Hudson, NY) through a scrotal incision and electrocautery under isoflurane anesthesia and aseptic conditions. The incision was closed with wound clips. For sham operation, the testes were gently drawn out but immediately placed back. The surgery protocol as well as those described below was approved by the Roswell Park Cancer Institute Animal Care and Use Committee. Spaying (ovariectomy) and sham operation in female C57BL/6N mice were performed by Taconic at 4 weeks of age.

### Generation of the UGT1A3 transgenic mice

To generate the UGT1A3 transgenic mice, we used the pLIVE™ (Liver *I**n* *V**ivo* Expression) vector purchased from Mirus Bio (Midison, WI), which is designed for high and liver-specific transgene expression in mice. This vector utilizes a chimeric promoter composed of the minimal mouse albumin promoter and the mouse alpha fetoprotein enhancer II. Two introns were also engineered into the plasmid (flanking the transgene) to further enhance high-level transgene expression. Human UGT1A3 cDNA was cloned to the pLIVE vector, generating pLIVE-UGT1A3. Briefly, the full-length human UGT1A3 coding sequence was amplified by PCR from the normal human liver cDNA using BamH I-forward (5′-CTGTCCGTGTCTTCTGCTGAG-3′) and Xho I-reverse (5′-TCAATGGGTCTTGGATTTGTG-3′). Amplified PCR products were digested by BamH I and Xho I endonucleases and ligated into pLIVE™ pre-digested with the same restriction enzymes. The construct was sequenced to ensure the correct sequence and orientation of the gene. Transient transfection of this plasmid into liver HepG2 cells resulted in robust expression of UGT1A3 (result not shown), confirming that the plasmid is highly functional in liver cells. The plasmid was digested by Bgl II and Sbf I restriction endonucleases, and the linearized 3.41 kb enhancer (Abl)-promoter (Afp)-transgene-polyA fragment was purified and micro-injected into mouse zygotes (C57BL/6N background). Two UGT1A3 transgenic founders were produced, as identified by PCR analysis of tail DNA, using oligonucleotide primers specific for human UGT1A3 as previously reported [34]. The hemizygous C57BL/6N-Tg(Alb/Afp-UGT1A3) founders were propagated by breeding with wild-type C57BL/6N mice from Taconic (Hudson, NY). As expected, 50% of F1 generation mice from each founder were UGT1A3-positive, but the F1 mice from one of the founders showed significantly higher UGT1A3 expression in the liver than those from the other founder (result not shown), as measured by RT-PCR. The F1 mice which expressed relatively high levels of UGT1A3 in the liver were interbred to generate homozygous C57BL/6N-Tg(Alb/Afp-UGT1A3) transgenic mice, abbreviated as UGT1A3+ mice, as well as non-transgenic littermates, which were used in the present study. The fertility and other parameters of the transgenic mice were indistinguishable from their wild type counterparts. There are 27 ± 5.2 (SD, *n* = 9) copies of UGT1A3 in these mice, as determined by qPCR by Charles River Genetic Testing Services (Troy, NY). The chromosomal location of the transgene and the orientation of transgene copies have not been determined.

### Animal treatment with ABP

ABP was dissolved in dimethyl sulfoxide and administered to mice by intraperitoneal injection in a volume of 2.5 μl/g body weight. The mice were killed 24 h post dosing, and relevant organs were removed rapidly, rinsed in ice-cold PBS, flash frozen in liquid nitrogen, and stored at –80°C until analysis.

### Measurement of tissue dG-C8-ABP

Sample preparation, including DNA isolation, quantification, enzymatic digestion and protein removal, and measurement of dG-C8-ABP by capillary liquid chromatography and nanoelectrospray ionization-tandem mass spectrometry have been previously described [35, 36].

### Measurement of tissue UGT activity

Sample preparation and measurement of enzymatic activity were performed as described previously [17] with minor modifications. Microsomes were used for measurement of ABP-specific UGT activity except for bladder. The microsome yield from each mouse bladder was very low; consequently, tissue homogenate supernatant cleared of tissue debris by low-speed centrifugation was used. UGT-catalyzed ABP glucuronidation was carried out in 0.2 ml of reaction volume for 15 min (zero order reaction) at 37°C, stopped by addition of 0.2 ml ice-cold methanol to each reaction, and then centrifuged at 16,000 g for 3 min at 4°C. To preserve the stability of ABP-G generated in the reaction, the supernatants were stored at –80°C before analysis by high performance liquid chromatography (HPLC). A Partisil™ 10 ODS-2 reverse phase column (4.6 × 250 mm) linked to an Agilent system (1100 series) with a diode-array detector was used. The mobile phase was comprised of 50 mM potassium phosphate at pH 6.8 and methanol, which was run at 1.75 ml/min, with methanol changed linearly from 35% to 55% over 0 to 7 min, 55% to 80% (7–15 min), 80% to 35% (15–16 min), and then kept at 35% (16–22 min) to re-equilibrate the column. The eluates were monitored at 280 nm, and peak area integration was performed via Agilent ChemStation software. APB-G standard was used to establish a calibration curve. Both ABP-G and APB were used as positive controls for the HPLC.

### RT-PCR of UGT1A3

Total RNA was isolated from each tissue using the RNeasy Mini kit (Qiagen, Valencia, CA). Genomic DNA was removed by treatment with TURBO DNase. RNA (0.5 μg total RNA per sample) was reverse transcribed into cDNA in a 25 μl reaction mixture, using the TaqMan Reverse Transcription Reagents kit (Invitrogen, Grand Island, NY). The reverse transcription reaction was performed at 25°C for 10 min, followed by heating at 48°C for 30 min and then 95°C for 5 min. Each PCR amplification was carried out in 20 μl volume, containing 10 μl of 2x GoTaq Master Mix (Promega, Madison, WI), 0.5–1 μl of the reverse-transcribed mixture (cDNA), and 0.25 μM each of specific forward and reverse primers. The PCR was carried out using the primer sequences and conditions described before [34]. The PCR products were analyzed by electrophoresis with 1% agarose gel and displayed by ethidium bromide staining under UV light.

### Statistical analysis

Two-tailed Student *t*-test was used for data analysis, with the significance threshold set at *P* value of < 0.05.
